# Visualising the Cardiovascular System of Embryos of Biomedical Model Organisms with High Resolution Episcopic Microscopy (HREM)

**DOI:** 10.3390/jcdd5040058

**Published:** 2018-12-15

**Authors:** Wolfgang J. Weninger, Barbara Maurer-Gesek, Lukas F. Reissig, Fabrice Prin, Robert Wilson, Antonella Galli, David J. Adams, Jacqueline K. White, Timothy J. Mohun, Stefan H. Geyer

**Affiliations:** 1 Division of Anatomy & MIC, Medical University of Vienna, 1090 Vienna, Austria; barbara.maurer-gesek@meduniwien.ac.at (B.M.-G.); lukas.reissig@meduniwien.ac.at (L.F.R.); stefan.geyer@meduniwien.ac.at (S.H.G.); 2 The Francis Crick Institute, London NW1 1AT, UK; Fabrice.Prin@crick.ac.uk (F.P.); rwilson@ebi.ac.uk (R.W.); tim.mohun@crick.ac.uk (T.J.M.); 3 Wellcome Trust Sanger Institute, Cambridge CB10 1SA, UK; antonella.galli@abcam.com (A.G.); da1@sanger.ac.uk (D.J.A.); jacqui.white@jax.org (J.K.W.)

**Keywords:** imaging, high resolution episcopic microscopy, HREM, embryo, phenotyping, episcopic, 3D, mouse, chick, developmental biology

## Abstract

The article will briefly introduce the high-resolution episcopic microscopy (HREM) technique and will focus on its potential for researching cardiovascular development and remodelling in embryos of biomedical model organisms. It will demonstrate the capacity of HREM for analysing the cardiovascular system of normally developed and genetically or experimentally malformed zebrafish, frog, chick and mouse embryos in the context of the whole specimen and will exemplarily show the possibilities HREM offers for comprehensive visualisation of the vasculature of adult human skin. Finally, it will provide examples of the successful application of HREM for identifying cardiovascular malformations in genetically altered mouse embryos produced in the deciphering the mechanisms of developmental disorders (DMDD) program.

## 1. Introduction

During gastrulation, embryos form a simple tubular heart. It pumps blood into a similarly simple and symmetrically arranged arterial system. During the following embryo periods and, to smaller extent even peri- and postnatally, both the heart and vascular system undergo extensive remodelling, which transforms them into complexly shaped and asymmetrically arranged organs.

The remodelling processes are driven and orchestrated by genetic and biomechanical factors, which interact in a strict temporal and spatial corset. Errors result in moderate to severe cardiovascular disorders but can potentially trigger abnormal formation and growth of all organ systems throughout the body.

Due to the central role the cardiovascular system plays in the embryonic, foetal and postnatal life, the genetic and biomechanical factors regulating normal cardiovascular development and the mechanisms underlying its pathologies are the focus of biomedical research [1,2,3,4,5,6,7]. For such research, a large number of tools for labelling active genes and gene products and for modifying or physically challenging their interplay during critical steps of development and growth were created and successfully established. Yet, all these tools produce effects that need to be properly visualised. Hence the existence of decent visualisation methods is at the core of cardiovascular research.

In particular visualisation and descriptive analysis of in situ distribution of gene expression information and of subtle morphologic defects caused by gene deletions, hemodynamic experiments or by alteration of the physical or chemical environment in embryos of model organisms remains a major challenge. The traditional imaging approach, which is careful analysis of series of two-dimensional (2D) histologic sections, is highly inefficient. This chiefly for two reasons: Firstly, embryos and their cardiovascular systems are of a complex three-dimensional (3D) arrangement, making it virtually impossible to comprehend the exact topology on the basis of two-dimensional (2D) information. Secondly, the production and examination of histologic sections is slow and time-consuming.

For coping with the drawbacks of conventional histological sections and section-based imaging methods [8,9,10], the last few decades have seen the development of large numbers of innovative 3D imaging technologies covering the meso- and microscopic range. The most promising are micro computed tomography (µCT)—even termed as virtual histology [11,12,13,14,15,16,17,18,19], micro-magnetic resonance imaging (µMRI) [20,21,22,23,24,25,26,27,28,29,30], optical projection tomography (OPT) [31,32,33,34,35,36,37], optical coherence tomography (OCT) [38,39,40,41], photoacoustic tomography (PAT) [40,42], serial block face microscopy [43,44], ultrasound microscopy [45,46,47,48,49], Episcopic 3D microscopy (Epi3D) [50], Episcopic Fluorescence Image Capturing (EFIC) [51,52] and High Resolution Episcopic Microscopy (HREM) [53,54,55,56]. A similar, episcopic approach is used for serial block-face electron microscopy (SBFSEM), which offers ultrastructural 3D information [57,58,59].

This paper aims to focus on one of these techniques, HREM, and its potential for visualising the cardiovascular system of normal and genetically altered embryos of biomedical models. In addition, it will briefly introduce the ability of using HREM for visualising the vascular system in biopsy material stemming from human tissue.

## 2. What Is High-Resolution Episcopic Microscopy (HREM)

High resolution episcopic microscopy is a technique for visualising the morphology of materials embedded in eosin stained methacrylate resin JB4. While cutting through the sample, series of inherently aligned digital images are created from subsequently exposed block surfaces. These images are virtually stacked and converted to volume data of high resolution and quality.

HREM volume data fit excellently for in situ visualisation, volumetry, planimetry and mathematical analysis of the components of the cardiovascular system of genetically normal, genetically engineered and physically and chemically challenged embryos of all popular model organisms. In selected cases HREM also permits 3D visualisation of gene expression and gene product patterns and the characterisation of cell distributions in their natural context. Hence, it was already successfully employed in several projects studying normal embryogenesis [60,61,62,63,64,65] and the role genes play in selected human diseases [66,67,68,69,70]. But, most importantly, HREM was introduced for the deciphering the mechanisms of the developmental disorders program (DMDD, www.dmdd.org.uk) as a method for screening the morphological phenotype of mouse embryos harvested at embryonic day (E) 14.5. In DMDD nearly 700 mouse embryos, stemming from 87 knock out lines, which produce embryonically lethal offspring were screened and their annotated data are provided on a freely accessible webpage [71,72,73,74].

The workflow of HREM data creation is simple and will be briefly outlined in the following chapters.

### 2.1. Sample Preparation

Sample preparation involves fixation and dehydration. A broad spectrum of fixation solutions and dehydration in series of increasing ethanols or methanols is suitable. Yet, for gaining excellent data quality, sample processing has to be slightly adapted to meet the specific requirements of tissues, organisms and developmental stages [53,75]. Also, if possible, perfusion with physiologic solutions for washing out the blood of cardiac chambers and/or blood vessels should be performed prior to embedding.

#### 2.1.1. Fixation

Most commonly, fresh material and loose neonatal and adult tissues are fixed in 4% PFA/PBS or Bouin’s solution for 24 h to several days, depending on sample size. Fixation time lasts for at least 1 day and does not exceed several weeks. Adult tissues, especially biopsy material harvested from the human skin and heart, are fixed in 2% PFA/PBS containing 4% carbolic acid and the samples are kept in this solution for at least one week and a maximum of twelve months [76]. If whole mount staining with dye reactions, e.g., the NBT/BCIP detection system was conducted prior to dehydration and resin embedding, the samples must be immersed in fixation solution before and after the staining procedure.

#### 2.1.2. Dehydration

After fixation, the samples are dehydrated in a series of methanols or ethanols with increasing concentrations (50%, 70%, 80%, 90%, 96%). Additional dehydration steps might be added for minimizing shrinkage artefacts. Depending on the volume of the specimens, they are kept from approximately 20 min (early zebrafish embryos) to up to 3 h (dense skin biopsy material) in each alcohol. Tissue contrast in large tissue samples profits from adding eosin to the higher alcohols [75,76].

#### 2.1.3. Infiltration

After dehydration, the samples are infiltrated with and embedded in JB4-resin (Polysciences) containing eosin (0.4 g/100 mL) or alternatively eosin and acridine orange (0.275 g eosin/100 mL and 0.055 g acridine orange/100 mL). Except for stirring eosin/arcridine orange into the solutions, the infiltration and embedding solutions are prepared following the instructions of the Polysciences embedding kit. Infiltration times depend on specimen size and range between 3–4 h (small zebrafish embryos) and 48 h (4 mm skin biopsy material).

#### 2.1.4. Embedding

Following infiltration, the specimens are placed in embedding moulds, filled with liquid embedding solution, composed of JB4-resin (Polysciences), which contains eosin (0.4 g/100 mL) or alternatively eosin and acridine orange (0.275 g eosin/100 mL and 0.055 g acridine orange/100 mL). The typical size of commercial embedding moulds ranges between 6 × 8 × 5 mm^3^ and 8 × 10 × 16 mm^3^.

After setting the block holders on top of the moulds, the moulds with block holders are sealed airproof with paraplast or by covering the surface with mineral oil to support hardening of the resin. Placing the airproof covered moulds in the fridge for approximately two days reduces the likeliness of the occurrence of bubbles or cavities inside the resin.

After hardening is completed, the polymerised blocks are either baked at 70–90 °C for 1 to 2 days for accelerated hardening and darkening of the resin, or simply stored for at least a week at room temperature. Blocks are then transferred to the room in which they are sectioned and stored for a few days before they are mounted on the microtome of the HREM apparatus. Storage for months is possible, although blocks older than one year tend to be brittle and break more easily during sectioning.

### 2.2. HREM Data Generation

The resin blocks are mounted in an HREM apparatus and sectioned, while series of digital images showing freshly cut block surfaces are captured. Sectioning is performed in an air-conditioned room at 18° to 20° Celsius, as high temperatures and humidity soften the block. The specifications of the components comprising a HREM-setup and the exact workflow during sectioning have been described in detail previously [56,77].

#### 2.2.1. HREM Apparatus

**Figure 1:**
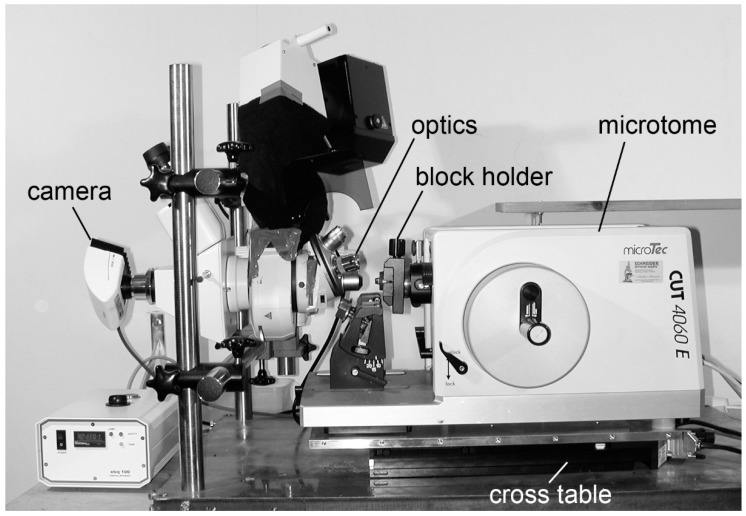
HREM apparatus.

An HREM apparatus essentially comprises a microtome or device for sectioning resin blocks in 1 to 5 µm steps. The sectioning device is equipped with a photo-position, at which the block containing the sample reproducibly stops after each cut. A fluorescence optics is aligned with the photo-position. Its optical axis comprises a GFP or YFP filter cube. If specimens are specifically stained with bluish stains, GFP or YFP and Texas Red filter cubes are subsequently placed in the optical axis. On the phototube of the optics sits a digital camera, which is connected to a PC operating a data generation software that orchestrates cutting, image capturing and image storage. A device for focusing and for adjusting the field of view completes the set up (Figure 1).

Various prototypes of HREM apparatuses are in use [53,78]. But recently a ready to use setup has also been made commercially available by Indigo Scientific Ltd. (Baldock, UK) (OHREM, http://www.indigo-scientific.co.uk/).

In routine projects, HREM apparatuses produce volume data comprised of 3000–4000 inherently aligned section images in about 8 h. The voxel dimensions of the created volume data range between 2 × 2 × 2 µm^3^ and 5 × 5 × 5 µm^3^, depending on the desired field of view. Larger and smaller dimensions are possible but require customised set ups and skilled operators.

#### 2.2.2. Data Processing

HREM data are either contrast enhanced by routines and archived, or directly loaded into 3D visualisation software, such as Amira^®^ (versions 5–6, ThermoFisher Scientific, Merignac, France), Osirix^®^ (www.osirix-viewer.com, versions 6–8, Pixmeo SARL, Bernex, Switzerland) or Imaris^®^ (version 9, Bitplane AG, Zürich, Switzerland). If section thickness does not correspond with the pixel size of the digital images, scaling of the single images to match the section thickness by using Photoshop^®^ (version 11, Adobe Systems, San Jose, CA, USA) or IrfanView (irfanview.com, version 4, Irfan Škiljan, Wiener Neustadt, Austria) routines is of advantage for later metric analysis.

#### 2.2.3. Data Visualisation

HREM data are ideal for immediate volume rendering and thus 3D models with exceptional resolution can be generated within seconds. The high contrasts of HREM data enable fully automated segmentation of body surfaces and subsequent surface rendering. 3D surface modelling of embryo organs, such as the central nervous system, lungs, liver pancreas, endocrine glands and others requires semi-automatic segmentation by, e.g., using region growing algorithms and interactive demarcation of the organ borders from adjacent structures. 3D surface modelling of structures such as blood vessels, nerves and ganglia requires manual tracing in most cases.

Rapid visualization of specifically labelled structures (e.g., LacZ or NBT/BCIP stained gene products) is possible by using multi-channel imaging. Two images are captured from each block surface. One with a GFP or YFP and one with a Texas Red filter set. Overall embryo morphology is visualised by using volume rendering in the GFP/YFP image set. Specifically stained structures are visualised as surface models after automated threshold segmentation in the image set captured with the Texas Red filter (Leica-Microsystems, Wetzlar, Germany).

## 3. Examples

Since its first publication in 2006, HREM has been used to analyse a variety of embryonic and adult samples. The following section provides examples of the successful use of HREM for analysing cardiovascular components.

Use of all animals was in accordance with UK Home Office regulations, the UK Animals (Scientific Procedures) Act of 1986 and approved by the Wellcome Trust Sanger Institute’s Animal Welfare and Ethical Review Body.

### 3.1. Skin Vasculature

The dermal layer of the skin harbours a highly complex system of blood vessels. They guarantee smooth supply of nutrients, oxygen and endocrine signals to all cells in all skin layers and, at the same time aid thermoregulation of the entire body.

**Figure 2:**
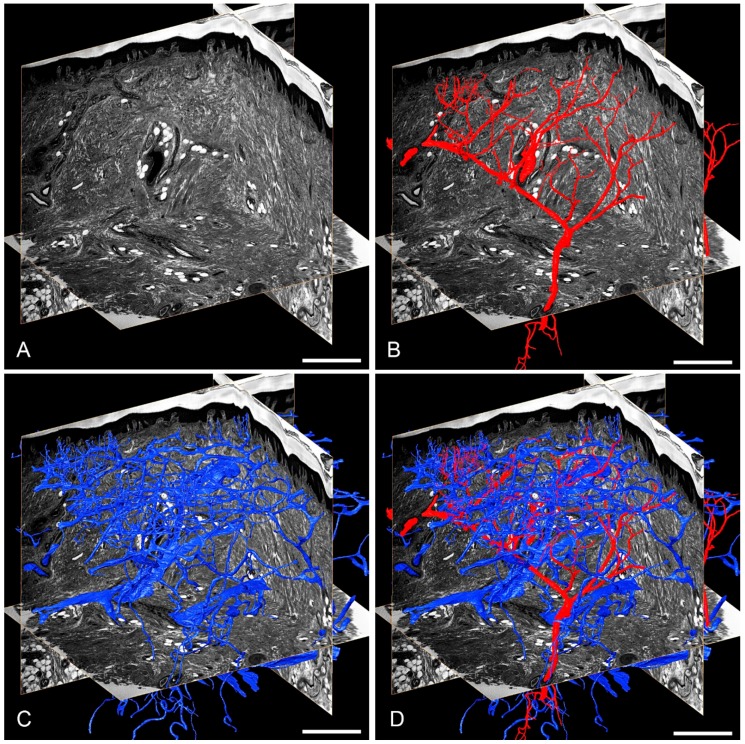
Thick skin of human thumb pad. Virtual sections through HREM data (**A**) together with surface models of dermal arteries (**B**), dermal veins (**C**) and both (**D**). Scalebars: 500 µm.

In a few anatomical studies, HREM proved to be highly useful for analysing the topology and architecture of the dermal arteries in various regions of the human skin [76,79,80]. Yet it was also successful in visualising neovascularisation in wound healing models [81,82,83] (Figure 2). In this context we like to emphasise that the dense tissues of adult humans and animals prevent automatic detection of blood vessels, where blood vessel segmentation has to be performed by manual tracing.

### 3.2. Biomedical Model Organisms

#### 3.2.1. Zebrafish and Frog Embryos

**Figure 3:**
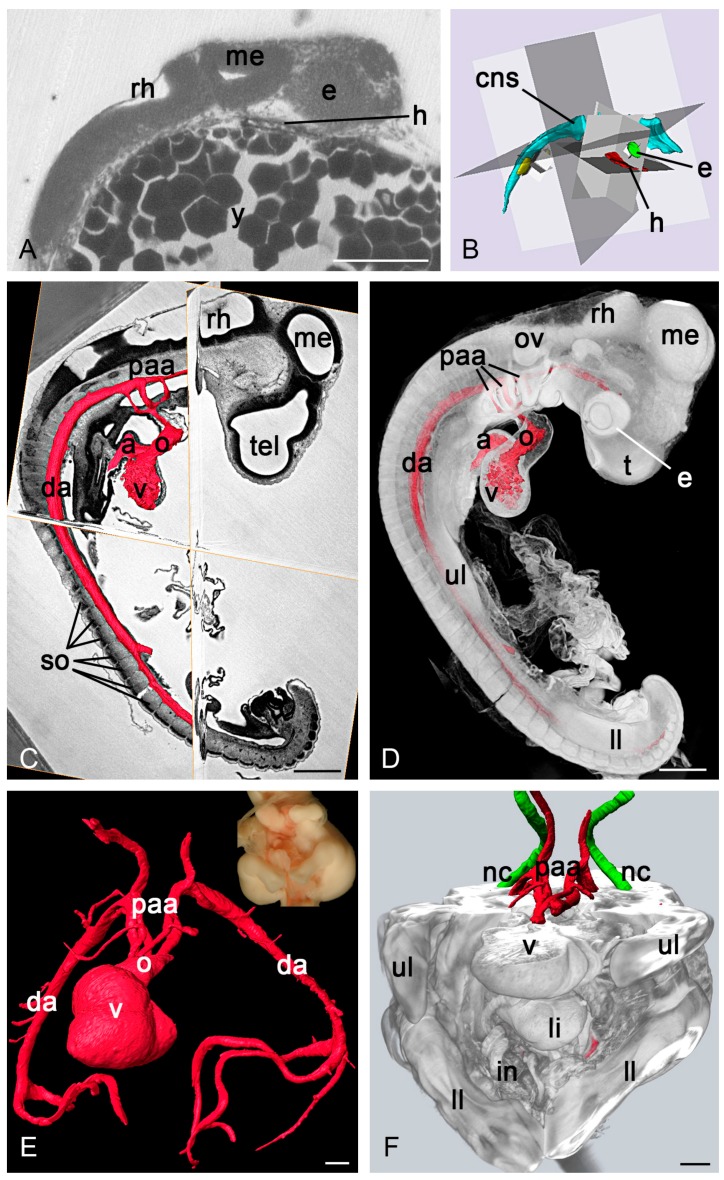
HREM for analysing the cardiovascular system of biomedical models. (**A**) Sagittal section through the head of a zebrafish embryo, 48 h after conception. (**B**) Surface models of the cranial parts of the central nerve system (cns), eye (e) and heart (h) with mathematically calculated and displayed planes through their main axes. (**C**,**D**) Surface models of the heart and arteries (red) of a chick embryo of stage 18 according to Hamburger Hamilton (HH) together with virtual resections through HREM data (**C**) and in the context of a semitransparent volume model of the embryo surface (**D**) respectively. (**E**,**F**) HH 34 chick embryo with cephalothoracopagus malformation. Surface models show a single heart and pharyngeal arch artery system (red) feeding the descending aorta of each twin (**E**). Additional surface models of the notochords (nc, green) in an opaque volume model of the surface with virtually excluded cranial parts from anterior (**F**). a, atrium; da, dorsal aorta; e, eye; in, intestine; li, liver; ll, lower limb; me, mesencephalon; nc, notochord; o, outflow tract; ov, otic vesicle; paa, pharyngeal arch arteries; rh, rhombencephalon; so, somite; t, telencephalon; ul, upper limb; v, ventricle. Scalebars: 100 µm (**A**), 500 µm (**C**–**F**).

HREM has been successfully used to image the heart of early zebrafish and frog embryos [53]. Due to the small sample size, also whole mount in situ hybridisation of *mlc2a* products could be used and allowed automated detection of heart cells. The insensitivity of HREM for artefacts caused by yolk and the high-resolution of the produced volume data allowed 3D imaging of the heart tube of 24–28 hpf zebrafish embryos in the context of the entire embryo sitting on the yolk sac. These data are excellently fitted for metric analysis of the cardiac axis of normal and mutated embryos in respect to the axis of the neural tube and other embryonic structures (Figure 3).

#### 3.2.2. Chick Embryos

HREM was successfully applied for visualising the heart and great blood vessels of normally developed and biomechanically challenged early to late chick embryos, from developmental stage 5 to 35 according to the staging system suggested by Hamburger and Hamilton [84] (Figure 3). The data enabled precise descriptions of pharyngeal arch artery remodelling and, in early embryos 3D visualisation of the distribution of specifically labelled *Tbx5* and *nkx2.5* products in the myocardium. In addition, HREM was successfully applied for visualising cardiovascular morphology of experimentally produced mutants [85,86] and for metric descriptions of the dimensions and angles of the great arteries [60,86].

In early embryos HREM permits visualisation of the cardiovascular system in the context of the whole embryo. Embryos older than HH28 must be parted for HREM imaging and only the thoraces or isolated hearts can be processed.

#### 3.2.3. Mouse Embryos

HREM proved to work excellently with normal and genetically engineered early to late mouse embryos. It was successfully used visualising the single steps during cardiovascular remodelling and for metric analysis of the dimensions of the great intrathoracic arteries and the lengths of vessel segments [60,62,63,87,88,89]. HREM also was applied for visualising structural abnormalities in various knock out lines serving as models for human diseases [68,90,91,92].

**Figure 4:**
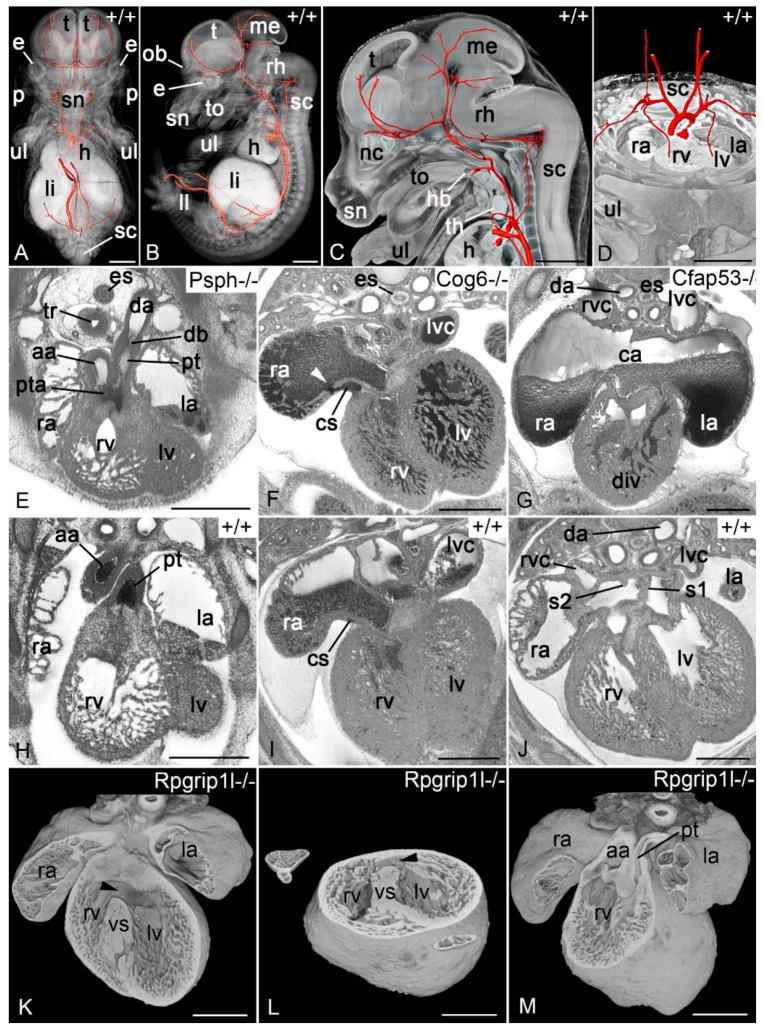
Cardiovascular features in HREM data of mouse embryos harvested at E14.5. (**A**,**B**) Surface rendered models of the arteries (red) together with a semi-transparent volume model from frontal (**A**) and lateral (**B**). (**C**) Surface rendered models of the head arteries (red) together with a sagittally sectioned volume model of the upper body half of an embryo. (**D**) Surface rendered models of the great intrathoracal arteries in an axially sectioned volume model. For details regarding artery topology see [87]. (**E**–**J**) Axial HREM sections, showing examples of cardiovascular malformations diagnosed in mutants produced in the DMDD program (**E**–**G**) and comparable sections through controls (**H**–**J**). Provided examples show persistent truncus arteriosus (pta) in a Psph mutant (**E**), additional opening of the coronary sinus (cs) into the right atrium (arrowhead) in a Cog6 mutant (**F**), and a complex malformation including common atrium (ca), double inlet left ventricle (div) and right sided descending aorta (da) in a Cfap53 mutant (g). (**K**–**M**) Volume rendered models of isolated hearts displaying heart malformations of a Rpgrip mutant. Perimembranous ventricular septal defect (pVSD) (**K**), muscular ventricular septal defect (mVSD) (**L**), double outlet right ventricle (DORV) (**M**). aa, ascending aorta; db, ductus arteriosus; e, eye; es, espophagus; h, heart; hb, hyoid bone; la, left atrial appendix; ll, lower limb; li, liver; lv, left ventricle; lvc, left vena cava superior; me, mesencephalon; nc, nasal cavity; ob, olfactory bulb; p, pinna; ra, right atrial appendix; rh, rhombencephalon; rv, right ventricle; rvc, right vena cava superior; sc, spinal chord; sn, snout; s1, septum primum; s2, septum secundum; t, telencephalon; th, thymus; to, tongue; tr, trachea; ul, upper limb; vs, ventricular septum. Scalebars 1 mm (**A**–**D**), 500 µm (**E**–**M**).

An important application of HREM was its use in the scope of the Deciphering the Mechanisms of Developmental Disorders (DMDD) program (https://dmdd.org.uk). HREM data of nearly 700 embryos of 87 knock out lines producing embryonically lethal offspring have been produced and thoroughly analysed in this program. Due to the unmatched spatial resolution and image quality of HREM and the availability of newly created reference data precisely defining normal cardiovascular morphology in the substages of E14.5 [93] vascular variations, large and small heart defects, but also entirely new and potentially life-threatening cardiovascular abnormalities were diagnosed and annotated [72] (Figure 4).
